# Alzheimer’s disease and the fornix

**DOI:** 10.3389/fnagi.2014.00241

**Published:** 2014-09-11

**Authors:** Kenichi Oishi, Constantine G. Lyketsos

**Affiliations:** ^1^The Russell H. Morgan Department of Radiology and Radiological Science, School of Medicine, Johns Hopkins UniversityBaltimore, MD, USA; ^2^Department of Psychiatry and Behavioral Sciences, Johns Hopkins Bayview and Johns Hopkins MedicineBaltimore, MD, USA

**Keywords:** Alzheimer’s disease, mild cognitive impairment, diffusion tensor imaging, deep brain stimulation, fornix, limbic system, magnetic resonance imaging, white matter

## Abstract

Alzheimer’s disease (AD) is the most common form of neurodegenerative dementia. Researchers have long been focused on the cortical pathology of AD, since the most important pathologic features are the senile plaques found in the cortex, and the neurofibrillary tangles and neuronal loss that begin in the entorhinal cortex and the hippocampus. In addition to these gray matter (GM) structures, histopathological studies indicate that the white matter (WM) is also a good target for both the early diagnosis of AD and for monitoring disease progression. The fornix is a WM bundle that constitutes a core element of the limbic circuits, and is one of the most important anatomical structures related to memory. Functional and anatomical features of the fornix have naturally captured researchers’ attention as possible diagnostic and prognostic markers of AD. Indeed, neurodegeneration of the fornix has been histologically observed in AD, and growing evidence indicates that the alterations seen in the fornix are potentially a good marker to predict future conversion from mild cognitive impairment (MCI) to AD, and even from cognitively normal individuals to AD. The degree of alteration is correlated with the degree of memory impairment, indicating the potential for the use of the fornix as a functional marker. Moreover, there have been attempts to stimulate the fornix using deep brain stimulation (DBS) to augment cognitive function in AD, and ongoing research has suggested positive effects of DBS on brain glucose metabolism in AD patients. On the other hand, disease specificity for fornix degeneration, methodologies to evaluate fornix degeneration, and the clinical significance of the fornix DBS, especially for the long-term impact on the quality of life, are mostly unknown and need to be elucidated.

## Introduction

Alzheimer’s disease (AD) research has targeted beta-amyloid and tau pathologies as diagnostic or progression markers. White matter (WM) shows less pathologic evidence of beta-amyloid or tau protein; nonetheless, alterations are seen in WM as well. Among WM bundles reportedly abnormal in AD, the fornix is especially interesting because robust and consistent alterations have been demonstrated. These could predict future gray matter (GM) atrophy and conversion from mild cognitive impairment (MCI) to AD, and even from cognitively normalcy to AD. Moreover, there are ongoing efforts to impact the biologic processes of AD by stimulating the fornix using deep brain stimulation (DBS). This review focuses on the fornix as a potential alternative diagnostic and prognostic marker, as well as a potential therapeutic target in AD.

### WM alterations seen in AD

Histopathological studies have revealed various types of WM alterations in AD. Symmetrical WM changes compatible with incomplete infarction, independent of the GM changes, have been reported. These WM changes are partly explained by co-existing small vessel disease (Brun and Englund, 1986; Englund et al., 1988). WM changes are also related to cerebral amyloid angiopathy of AD, supporting a vascular contribution to the WM alterations (Haglund and Englund, 2002). Chronic hypoxia caused by vascular alterations might induce oxidative stress that could damage axonal membranes and myelin, and the reactivity of oligodendroglia to such damage (Back et al., 2011). However, in preclinical AD, WM alterations are independent of vascular alterations (de la Monte, 1989). In addition, degeneration of the WM was more obvious than that of the GM in early stages (de la Monte, 1989), suggesting that WM alteration precedes GM pathology. Indeed, the earliest disease-related anatomical changes have been observed in the axons and dendrites of AD animal model (Gunawardena and Goldstein, 2001; Pigino et al., 2003; Stokin et al., 2005; Chevalier-Larsen and Holzbaur, 2006). Therefore, mechanisms that cause the early pathological changes, aside from vascular alterations, might exist. According to the updated amyloid cascade hypothesis (Hardy and Selkoe, 2002), one of the high upstream events is production of soluble beta-amyloid oligomers, been discovered to be toxic to WM structure and function (Lue et al., 1999; Xu et al., 2001; Lee et al., 2004). This could be a major cause of the primary WM alterations in AD (Roher et al., 2002; Chalmers et al., 2005) at least in early stages.

In addition to widespread WM alterations, structure-specific WM alterations have been observed, especially in limbic structures (Damoiseaux et al., 2009; Acosta-Cabronero et al., 2010; Smith et al., 2010; Liu et al., 2011). This might at least partly be explained by aberrant axonal transport due to misregulation of tau seen in early disease stage (Alonso et al., 1994; Ebneth et al., 1998; Dawson et al., 2010), with resultant tau aggregation that is closely related to neurodegeneration (Braak and Braak, 1995). Since the neurodegeneration of AD usually propagates systematically from the transentorhinal area to limbic structures, to neocortex at the most advanced stage, associated Wallerian-like degeneration (Bossy-Wetzel et al., 2004; Coleman, 2005) likely follows the same order, which might explain structure-specific WM alterations in AD (Bramblett et al., 1992).

Congruent with histopathological findings, WM alteration has been observed *in vivo* using various imaging modalities. Among these, diffusion tensor imaging (DTI) is one of the most effective modalities for the investigation of the WM anatomy since DTI has the ability to visualize WM fibers, based on directionality, and has the capability to provide detailed information about microscopic organization of the fibers. A meta-analysis of DTI studies indicated that WM alterations in AD are widespread throughout the brain (Sexton et al., 2011), particularly in limbic fibers, direct connections to the medial temporal lobe and the most vulnerable WM structures (Rose et al., 2000; Kantarci et al., 2001; Medina et al., 2006; Ringman et al., 2007; Stahl et al., 2007; Zhang et al., 2007; Zhou et al., 2008; Damoiseaux et al., 2009; Mielke et al., 2009; Salat et al., 2010). WM damage quantified by DTI has been correlated with atrophy in anatomically connected GM areas in AD patients, but the correlation was not robust in patients with amnestic MCI (Agosta et al., 2011). These findings support the histopathological observation that primary WM alterations may precede both GM degeneration, as well as secondary degenerative processes of the WM after neuronal loss. Based on the evidence about WM pathology in AD, Sachdev et al. proposed incorporating WM alterations into the model (Jack et al., 2010) of the pathophysiological cascade in AD (Sachdev et al., 2013).

### Why is the fornix important?

The fornices are WM bundles that originate from the bilateral hippocampi, merge at the midline of the brain, again divide into the left and right side, then into precommissural and postcommissural fibers, terminating at the septal nuclei, nucleus accumbens (precommissural fornix), and hypothalamus (postcommissural fornix). The fornix constitutes a core element of the limbic circuit that is vulnerable in AD, and one of the most important anatomical structures related to episodic memory, impairment of which is an initial symptom of AD (Hopper and Vogel, 1976; Mehraein and Rothemund, 1976). The fornix is also related to the cholinergic dysfunction characteristic of AD (Milner and Amaral, 1984; Wenk et al., 1987; Ransmayr et al., 1989; Sara, 1989; Schegg et al., 1989; Bunce et al., 2003; Colom et al., 2010). These functional and anatomical features have naturally captured researcher attention seeking diagnostic and prognostic markers of AD. Indeed, alterations of the fornix, such as demyelination or axonal loss, were reported as early as in 1976 (Hopper and Vogel, 1976) and have been consistently identified in AD (Ringman et al., 2007; Teipel et al., 2007; Mielke et al., 2009; Acosta-Cabronero et al., 2010; Salat et al., 2010; Liu et al., 2011; Douaud et al., 2013).

The other important anatomical feature of the fornix, compared to other WM areas, is its location. The body of the fornix is readily identifiable in the mid-sagittal plane of a brain MRI, without other WM structures adjacent to it. For research using brain MRI, this feature is particularly important. The thickness of the fornix can be easily measured on structural MRI, using T1- and T2-weighted images. Generally, identification of the boundaries of WM structures on structural MRI is not easy because the boundaries between adjacent WM structures are often invisible: the fornix is one of several exceptions. DTI is a good choice with which to identify the boundaries of WM bundles. Although a drawback of DTI is susceptibility to B0 distortion, especially for areas close to air cavities (e.g., nasal and oral cavities, frontal, ethmoid, sphenoid, and maxillary sinuses, and mastoid antrum), the fornix is less affected because there is a sufficient distance from the fornix to these air cavities. The anatomical location is also attractive from a neurosurgical point of view, since the fornix is surgically accessible for implanting electrodes for DBS (see Section Treatment), while other limbic structures are not easily accessible.

## Imaging study of the fornix in AD

### Region-of-interest studies

Atrophy of the fornix measured using T1-weighted MRI has often been reported in AD (Callen et al., 2001; Copenhaver et al., 2006), although the onset of this atrophy has been a subject of debate. Fletcher et al. (2013) reported that atrophy of the fornix is a strong predictor of conversion from normal cognition to MCI or AD, suggesting that atrophy is present in the fornix at an early disease stage, before clinical manifestations. Abnormalities in the fornix have also been identified using DTI. A reduction of fractional anisotropy (FA) and an increase in diffusivity in the fornix is a robust and consistent finding in AD as demonstrated with manual ROI- analysis (Ringman et al., 2007; Mielke et al., 2009; Oishi et al., 2012), and the extent of the changes in DTI-derived parameters have been correlated with cognitive decline (Mielke et al., 2009; Oishi et al., 2012). Notably, FA reduction was already present in an asymptomatic gene carrier of familial AD who went on to develop AD in the future (Ringman et al., 2007). Longitudinal observation of cognitively normal elderly or individuals with amnestic MCI indicated that reduced fornix FA predicts conversion from normal cognition to amnestic MCI and from amnestic MCI to AD (Oishi et al., 2012). Moreover, lower fornix FA predicted later cognitive decline and hippocampal atrophy (Mielke et al., 2012). These studies suggest that volume loss and FA reduction of the fornix is one of the earliest anatomical changes in AD that happens before clinical manifestations.

### Whole-brain studies

While region-of-interest analysis is a good choice when there are specific hypotheses (e.g., the fornix is affected in AD), the drawback is the hypothesis dependency. To evaluate the spatial specificity of these findings, whole-brain analysis is a good choice. Voxel-based analysis is one of the most widely used approaches for whole-brain analysis, and has been used to evaluate regional abnormalities in WM volume (Li et al., 2008; Balthazar et al., 2009; Guo et al., 2010; Serra et al., 2010; Yoon et al., 2011) or in DTI-derived parameters, such as anisotropy and diffusivity (Head et al., 2004; Medina et al., 2006; Xie et al., 2006; Teipel et al., 2007; Zhang et al., 2009). Although cross-sectional group comparisons have identified widespread WM abnormalities in AD, the above studies failed to identify reduced volume or FA in the fornix, except for a study that used multivariate analysis (Teipel et al., 2007). The main explanation is probably inaccuracy in image normalization: the accuracy of automated non-linear registration used in these studies was limited for thin structures like the fornix, in which only a few pixels of misregistration cause significant loss of sensitivity to detect differences between groups (Oishi et al., 2009). A common approach to solve this problem is to apply Tract-Based Spatial Statistics (TBSS; Smith et al., 2006), in which WM tracts are “skeletonized” to summarize the anatomy of WM structures, from which statistics are calculated. Since the scalar values perpendicular to the skeleton are projected onto the skeleton, the effect of WM misregistration is largely ameliorated. Most of the whole brain TBSS studies applied to AD have, indeed, identified an FA reduction in the fornix, as well as in other limbic fibers, even in early-symptomatic patients or in individuals at high risk for developing AD (Damoiseaux et al., 2009; Honea et al., 2009; Stricker et al., 2009; Zarei et al., 2009; Acosta-Cabronero et al., 2010; Bosch et al., 2010; Smith et al., 2010; Liu et al., 2011; Douaud et al., 2013).

**Figure 1:**
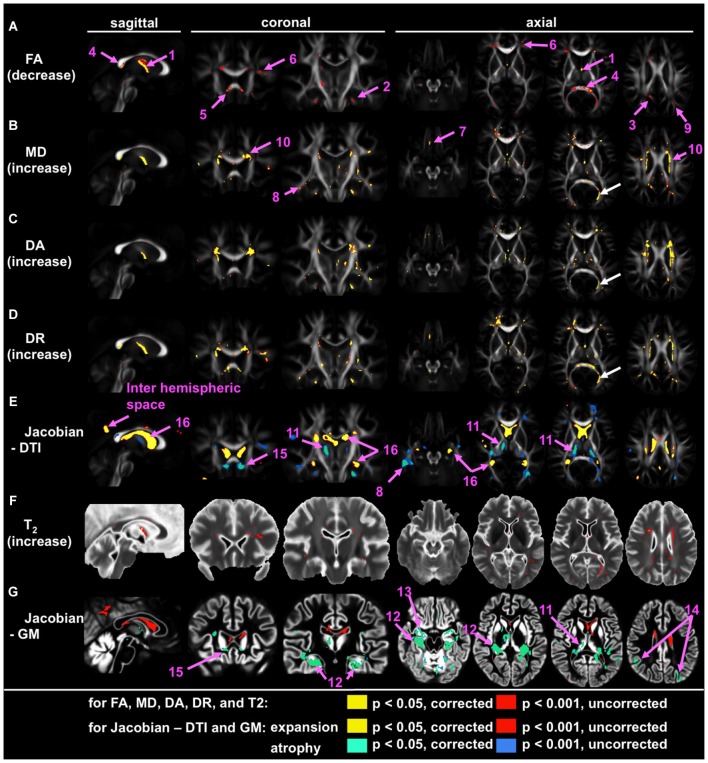
**Voxel-based group comparison between 19 AD and 22 cognitively normal age-matched control participants (NC), indicating the fornix as the hotspot**. Areas with signal or volume alterations in AD compared to NC are shown as colored maps, overlaid on an averaged FA map **(A–E)**, an averaged T_2_ map **(F)**, and an averaged GM segmentation map **(G)**. **(A)** Areas with reduced FA. **(B)** Areas with increased MD. **(C)** Areas with increased λ_‖_. **(D)** Areas with increased λ_⊥_. **(E)** Areas with an increased and decreased Jacobian, which was calculated from a transformation matrix obtained from the normalization of DTI. **(F)** Area with increased T_2_. **(G)** Areas with increased and decreased Jacobian, which were calculated from a transformation matrix obtained from the normalization of a GM segmentation map. Pink arrows with numbers indicate: 1 = the fornix; 2 = the cingulum; 3 = the posterior cingulate gyrus white matter; 4 = splenium of the corpus callosum; 5 = genu of the corpus callosum; 6 = the prefrontal white matter; 7 = the orbitofrontal white matter; the temporal white matter; 9 = the parietal white matter; 10 = the periventricular area; 11 = the thalamus; 12 = the hippocampus; 13 = the entorhinal area; 14 = the parietal cortex; 15 = the area between the caudate head and the gyrus rectus; 16 = the lateral ventricle. White arrows show the misregistration seen in the left posterior horn of the lateral ventricle (From Oishi et al., 2011a; with permission).

Application of large deformation diffeomorphic metric mapping (LDDMM) to DTI is another way to increase the sensitivity of whole-brain analysis, by which registration accuracy of the WM bundles could be increased. By using LDDMM, significant volume loss, FA reduction, and increases in mean diffusivity (MD) and radial diffusivity were detected in the fornix of individuals with AD (Oishi et al., 2011a,b). Figure 1 is an example of such an analysis, designed to find brain areas with AD-specific WM alterations. This analysis indicates significant FA reduction in the fornix, the splenium of the corpus callosum, as well as in several small areas in superficial WM in the frontal lobes. To further increase sensitivity, multivariate models (Ashburner and Kloppel, 2011), such as principal component analysis (PCA; Teipel et al., 2007), or to apply voxel-grouping methods, such as atlas-based analysis (ABA; Oishi et al., 2009), were applied: all of these detected abnormalities in the fornix in AD. These whole-brain studies using sophisticated approaches consistently indicate the fornix as the “hotspot”, in which the earliest anatomical changes begin (“Fornix First” (Landhuis, 2013)), and where the most robust changes are seen in AD.

### Pitfalls and issues in fornix image analysis

#### Misregistration

As mentioned previously, TBSS is currently a standard method by which to ameliorate WM tract misregistration. However, in patients with ventricular enlargement, like AD, the fornix is strongly deformed by the elevated and stretched corpus callosum, which results in uncorrectable misregistration, even with TBSS (Hattori et al., 2012). Therefore, careful interpretation is needed for AD studies. An inverse-projection of TBSS results onto original images is a simple method with which to identify invalid results caused by misregistration.

One solution to reduce the impact of erroneous mapping of voxels, and the consequent mis-parcellation of target structures, is to adopt multi-atlas approaches. Rather than using a single atlas for anatomical labeling, this approach uses multiple atlases to compute the likelihoods of labels, which are then fused to create an anatomical parcellation map for each image. This approach, which accounts for variations in brain anatomy, has been adapted to parcellate WM structures in DTI (Tang et al., 2014) with high accuracy even for individuals with ventricular enlargement. Applicability of the multi-atlas label fusion method to accurately define the fornix is a promising approach, which needs to be developed in the future with automated image analysis of AD.

#### Contamination of signal from cerebrospinal fluid

The fornix is prone to partial volume effects caused by contamination of signal from the cerebrospinal fluid (CSF), since it is a thin bundle that runs through the lateral ventricles that are filled with CSF. The effect is especially seen in individuals with AD because of atrophy, and thus, increased CSF signal in each voxel (Pfefferbaum and Sullivan, 2003; Metzler-Baddeley et al., 2012; Berlot et al., 2014). The greater diffusivity in CSF relative to the WM structures leads to false elevation of diffusivity values and a false reduction in FA values (Alexander et al., 2001; Vos et al., 2011). Therefore, reduced FA or increased diffusivity identified in the DTI studies of AD probably reflects pathological alterations of the fornix itself plus the partial volume effects caused by atrophy. Although this dual effect (atrophy + microstructural change) on the DTI parameters might make DTI a sensitive tool with which to detect early anatomical changes related to AD, it complicates inferences about the underlying anatomical changes. There have been several attempts to ameliorate the effects of CSF contamination in order to draw inferences about the microstructural changes in the WM structures, such as CSF-suppressed diffusion imaging (Kwong et al., 1991), or the free water elimination and mapping method that is applicable at the post-acquisition stage (Pasternak et al., 2009), both of which could detect alteration of the fornix in AD (Kantarci et al., 2010; Fletcher et al., 2014). Investigations of underlying changes in WM structures seem to suggest a good indication for the use these advanced methods (Metzler-Baddeley et al., 2012).

#### Cause of fornix degeneration and DTI parameters of choice

Since the contribution of each factor (see WM alterations seen in AD**)** to the degeneration of the fornix might vary depending on the degree of disease progression, longitudinal observation of the histopathology, from the preclinical to the advanced stages, is especially important. Use of the AD model in animals is an option for such longitudinal observation. A reduced size of the fornix is seen in the PDAPP mice (Gonzalez-Lima et al., 2001) and transgenic APP/PS1 mice (Delatour et al., 2006), and immunohistochemistry shows the presence of increasing amyloidosis and related microgliosis and astrogliosis (Maheswaran et al., 2009). However, longitudinal changes in the size of the fornix differ depending on the type of AD model mice. Since type and variations in gene mutation are relevant to the pathophysiology of AD model animals, and considerable differences are seen between human AD and AD model animals, human *in vivo* neuroimaging studies are important to understand the pathophysiological underpinnings. For early AD, diffusion measures (MD and axial and radial diffusivities) could detect the widespread pathology of AD more sensitively than FA (Acosta-Cabronero et al., 2010; Oishi et al., 2011a). However, for the fornix, there seems to be a tendency that a decrease in FA is more sensitive than an increase in diffusivity measures. Whether there is a difference in pathophysiology between the fornix and other WM areas remains to be elucidated.

## Clinical applications of the findings seen in the fornix

### Diagnosis and prediction

**Figure 2:**
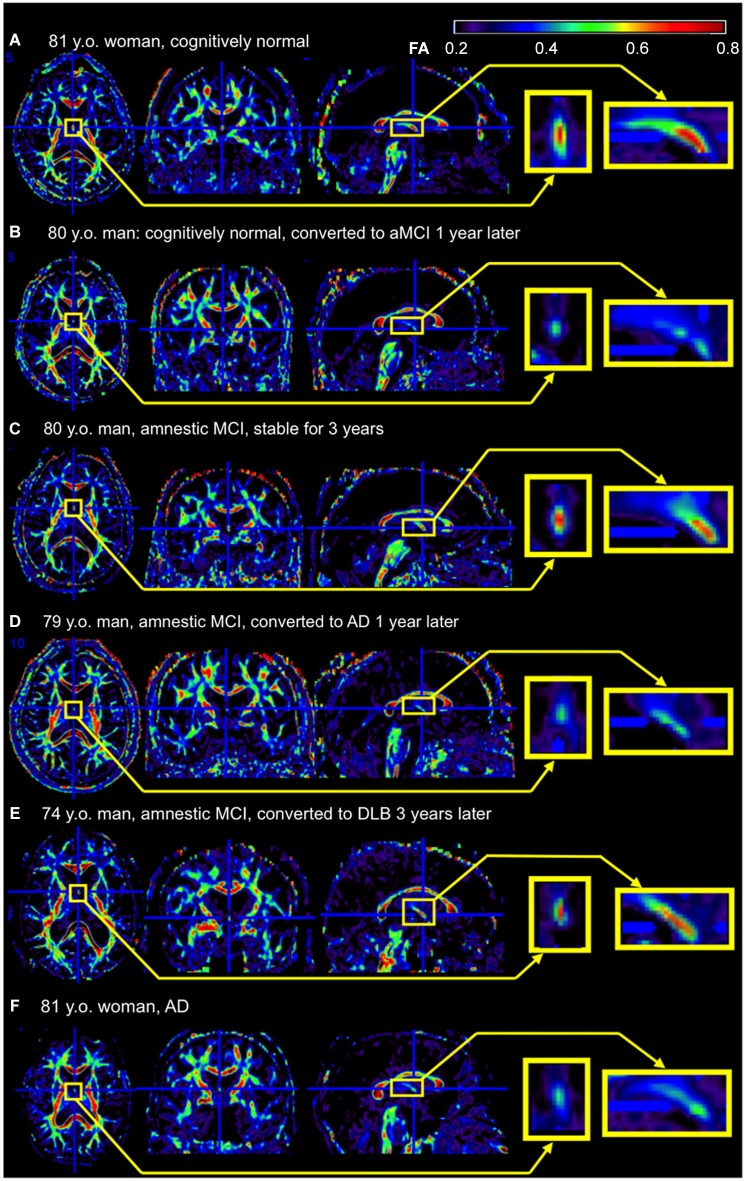
**Example of the fornix sign**. The axial (left), coronal (middle), and sagittal (right) slices of the color-scaled FA map are shown with the magnified view of the fornix (yellow rectangle). **(A)** A cognitively normal 81-year-old woman without the fornix sign. The core part of the fornix appears yellow to red (FA 0.5–0.8). **(B)** A cognitively normal 80-year-old man with the fornix sign. The fornix appears green (FA < 0.5). He converted to amnestic MCI (aMCI) 1 year after the scan. **(C)** An 80-year-old man with aMCI without the fornix sign. He was stable during 3 years observation period after the scan. **(D)** A 79-year-old man with aMCI with the fornix sign. He converted to AD 1 year after the scan. **(E)** A 74-year-old man with aMCI without the fornix sign. He converted to dementia with Lewy bodies 3 years after the scan. **(F)** An 81-year-old woman with AD with the fornix sign. FA, fractional anisotropy.

There are several hurdles when applying research findings to clinical practice. Research studies are based on selected participants with strict inclusion and exclusion criteria, and usually age- and gender-matched participants are recruited as controls. However, routine image-based diagnosis is almost exclusively performed by qualitative visual inspection, which is applied to each individual for clinical decision-making. Although numerous publications link AD to degeneration in the fornix, volume or the diffusivity are not quantified in daily practice because of the time-consuming nature of this process, and the inconsistent quantification methods used in research. The fornix sign, a discoloration of the fornix observed on color-coded FA maps of AD (Oishi et al., 2012), is one attempt to define qualitative image signs applicable to clinical images (Figure 2). Although sensitivity was limited, specificity was high. The positive likelihood ratio differentiating AD from controls and hence the ability to predict conversion of cognitively normal individuals to amnestic MCI, or from amnestic MCI to AD, was strong. However, further efforts are needed to identify appropriate scan parameters, cut-off values for the diagnosis and prediction, as well as to investigate degeneration in the fornix seen in other types of dementia.

Given that individuals with different types of memory complaints visit clinics, and physicians need to identify the etiology of the memory complaint for medical decision-making, the value of imaging signs that can separate AD from individuals with normal cognition are limited. Rather, clinically relevant imaging signs must provide clues that can be used to separate AD from other diseases that cause memory impairment. Indeed, the fornix might be affected by tumors, infections, inflammations (Yamamoto et al., 1990), metabolic abnormalities, vascular diseases (Molino et al., 2014), malformations, trauma, and hydrocephalus (Hattori et al., 2012) (for review, Thomas et al., 2011). Psychiatric diseases, such as schizophrenia and bipolar affective disorder (Oertel-Knöchel et al., 2014), or medical conditions, such as heart failure (Wu et al., 2007), also cause damage in the fornix. Less is known about the involvement of the fornix in other neurodegenerative dementias, such as frontotemporal dementia (FTD), dementia with Lewy bodies (DLB), and corticobasal degeneration (CBD). A study indicated substantial FA reduction in the fornix of the behavioral variant FTD that correlated with memory decline (Hornberger et al., 2012), and other studies have indicated that the fornix is affected in Pick’s disease, FTD with tau mutation, or TAR-DNA-binding protein-43 type C pathology, but not for FTD caused by a progranulin mutation or with fused-in-sarcoma protein accumulation (Rohrer et al., 2011; Hornberger et al., 2012). Changes in the fornix were not detected by group comparison between DLB and control groups (Kantarci et al., 2010), or between CBD and control groups (Rohrer et al., 2011), but other research has reported reduced FA in the fornix of some DLB patients, especially for those with hippocampal atrophy (Firbank et al., 2011). The appropriate clinical use of imaging modalities for the evaluation of the fornix is, therefore, an important future direction.

### Treatment

Inspired by neuroimaging evidence about early impairment of the limbic circuit seen in AD, and an unexpected augmentation of recollection during the hypothalamic/fornix DBS performed on a patient with obesity (Hamani et al., 2008), DBS of the fornix has been proposed as a novel therapy for AD to improve the neuronal circuitry involved in memory (Laxton et al., 2010). Based on the hypothesis that an important aspect of AD is that it is a system-level disorder affecting several integrated pathways related to memory and cognition (Lyketsos et al., 2012), and supported by rodent studies indicating that DBS improved memory and produced hippocampal neurogenesis (Toda et al., 2008; Hamani et al., 2011), a clinical trial evaluating the effect of DBS targeting the fornix is ongoing. Electrodes are placed adjacent to the fornix for high frequency electronic stimulation, with the hope of an immediate effect (direct effects of stimulating the fornix) and a disease-modifying effect (neurogenesis). Although its number of participants was small, the earlier Phase 1 trial suggested improved glucose metabolism in the posterior cortical areas, especially in cognitively improved participants after fornix DBS (Laxton et al., 2010; Smith et al., 2012). The assessment of long-term impact on cognitive function, activities of daily living, quality of life, and mortality is an important future direction.

## Conflict of interest statement

Constantine G. Lyketsos has received support from the following organizations Associated Jewish Federation of Baltimore, Weinberg Foundation, Forest, Glaxo-Smith-Kline, Eisai, Pfizer, Astra-Zeneca, Lilly, Ortho-McNeil, Bristol-Myers, and Novartis. Constantine G. Lyketsos has served as a consultant/advisor for Astra-Zeneca, Glaxo-Smith-Kline, Eisai, Novartis, Forest, Supernus, Adlyfe, Takeda, Wyeth, Lundbeck, Merz, Lilly, and Genentech. Constantine G. Lyketsos has received honorarium or travel support from Pfizer, Forest, Glaxo-Smith-Kline, and Health Monitor. This arrangement has been approved by the Johns Hopkins University in accordance with its conflict of interest policies.
